# Guillain–Barré syndrome spectrum associated with COVID-19: an up-to-date systematic review of 73 cases

**DOI:** 10.1007/s00415-020-10124-x

**Published:** 2020-08-25

**Authors:** Samir Abu-Rumeileh, Ahmed Abdelhak, Matteo Foschi, Hayrettin Tumani, Markus Otto

**Affiliations:** 1 Department of Neurology, Ulm University Hospital, 89070 Ulm, Germany; 2 Department of Neurology and Stroke, University Hospital of Tübingen, 72076 Tübingen, Germany; 3 Hertie Institute of Clinical Brain Research, University of Tübingen, 72076 Tübingen, Germany; 4 Neurology Unit, S. Maria delle Croci Hospital–AUSL Romagna, ambito di Ravenna, 48121 Ravenna, Italy; 5 Specialty Hospital of Neurology Dietenbronn, 88477 Schwendi, Germany

**Keywords:** COVID-19, SARS-CoV-2, Coronavirus, Guillain–Barré syndrome, Miller Fisher syndrome, Neurology, Autoimmune, Polyradiculopathy, Neuroimmunology

## Abstract

Since coronavirus disease-2019 (COVID-19) outbreak in January 2020, several pieces of evidence suggested an association between the spectrum of Guillain–Barré syndrome (GBS) and severe acute respiratory syndrome coronavirus-2 (SARS-CoV-2). Most findings were reported in the form of case reports or case series, whereas a comprehensive overview is still lacking. We conducted a systematic review and searched for all published cases until July 20th 2020. We included 73 patients reported in 52 publications. A broad age range was affected (mean 55, min 11–max 94 years) with male predominance (68.5%). Most patients showed respiratory and/or systemic symptoms, and developed GBS manifestations after COVID-19. However, asymptomatic cases for COVID-19 were also described. The distributions of clinical variants and electrophysiological subtypes resemble those of classic GBS, with a higher prevalence of the classic sensorimotor form and the acute inflammatory demyelinating polyneuropathy, although rare variants like Miller Fisher syndrome were also reported. Cerebrospinal fluid (CSF) albuminocytological dissociation was present in around 71% cases, and CSF SARS-CoV-2 RNA was absent in all tested cases. More than 70% of patients showed a good prognosis, mostly after treatment with intravenous immunoglobulin. Patients with less favorable outcome were associated with a significantly older age in accordance with previous findings regarding both classic GBS and COVID-19. COVID-19-associated GBS seems to share most features of classic post-infectious GBS and possibly the same immune-mediated pathogenetic mechanisms. Nevertheless, more extensive epidemiological studies are needed to clarify these issues.

## Introduction

Coronavirus disease 2019 (COVID-19) pandemic has rapidly spread around the world from Jan-2020, with more than 14,000,000 cases confirmed so far [1]. Although primary affecting the respiratory system, central and peripheral neurological manifestations associated with severe acute respiratory syndrome coronavirus 2 (SARS-CoV-2) infection have been increasingly reported [2–4]. In detail, several pieces of evidence suggested an association between SARS-CoV-2 infection and the development of Guillain–Barré Syndrome (GBS) [5–56].

GBS represents the most common cause of acute flaccid paralysis [57]. The classic form is an immune-mediated acute-onset demyelinating polyradiculoneuropathy (acute inflammatory demyelinating polyneuropathy—AIDP) typically presenting with ascending weakness, loss of deep tendon reflexes, and sensory deficits. Diagnosis of GBS relies on the results of clinical, electrophysiological, and cerebrospinal fluid (CSF) examinations (classically albuminocytological dissociation) [57–59]. The clinical spectrum of GBS encompasses a classic sensorimotor form, Miller Fisher syndrome (MFS), bilateral facial palsy with paraesthesia, pure motor, pure sensory, paraparetic, pharyngeal–cervical–brachial variants, polyneuritis cranialis (GBS–MFS overlap), and Bickerstaff brainstem encephalitis [57–60]. As regard electrophysiological features, three main subtypes are recognized: AIDP, acute motor axonal neuropathy (AMAN), and acute motor sensory axonal neuropathy (AMSAN) [57, 58, 61]. Peripheral nerve damage is thought to be provoked by an aberrant immune response to infections, in some cases driven by the production of autoreactive antibodies (anti-ganglioside antibodies) [57–59]. Potential triggering pathogens include both viruses [e.g., cytomegalovirus (CMV), Epstein–Barr virus (EBV), influenza virus, hepatitis E virus, and Zika virus] and bacteria (e.g., *Campylobacter Jejuni, Mycoplasma Pneumoniae*) [57, 58, 62]. However, a relationship with other events has been also described (e.g., vaccinations, surgery, administration of checkpoint inhibitors, and malignancy) [57, 58]. Given that a potential causal association with beta-coronaviruses [Middle East Respiratory Syndrome (MERS-CoV)] has already been speculated, the relationship between COVID-19 and GBS deserves undoubtedly further attention [63, 64].

With this background, our systematic review aimed to provide a comprehensive and updated overview of all case reports and series of COVID-19-related GBS to identify predominant clinical, laboratory, and neurophysiological patterns and to discuss the possible underlying pathophysiology.

## Methods

We performed a systematic review according to the SALSA (Search, Appraisal, Synthesis, and Analysis) analytic framework [65]. We screened in PubMed and Google Scholar databases for all case descriptions of GBS associated with COVID-19 that were published from January 1st 2020 up to July 20th 2020. Keywords (including all commonly used abbreviations of these terms) used in the search strategy were as follows: [“acute autoimmune neuropathy” OR “acute inflammatory demyelinating polyneuropathy” OR “acute inflammatory demyelinating polyradiculoneuropathy,” OR “acute inflammatory polyneuropathy” OR “Demyelinating Polyradiculoneuropathy” OR “Guillain–Barre Syndrome” OR “Guillain–Barre” OR ““Miller–Fisher” OR “Bickerstaff encephalitis” OR “AIDP” OR “AMAN” OR “AMSAN” OR polyneuritis cranialis] AND [“COVID-19” OR “Wuhan coronavirus” OR “novel coronavirus” OR “novel coronavirus 2019” OR “SARS” OR “SARS-CoV-2”]. Suitable references were also identified in the authors’ archives of scientific literature on GBS. We restricted our search to studies published in English, Spanish, or Italian. Publications that were not peer-reviewed were excluded from this study. PRISMA criteria were applied. For each case, we extracted data concerning demographic and clinical variables, results of diagnostic investigations, and outcome. If the GBS clinical variant [57] or the electrophysiological subtype [61] was not explicitly reported in the paper, we reconstructed it, when possible, from reported details. We also classified the diagnostic certainty of all cases according to the Brighton Criteria [66]. Searches were performed by SAR, AA, and MF. The selection of relevant articles was shared with all authors.

For statistical analysis, we used IBM SPSS Statistics version 21 (IBM, Armonk, NY, USA). Based on the distribution of values, continuous data were expressed as mean ±  standard deviation or as  median and interquartile range (IQR). Depending on the number of groups and data distribution, we applied the t test, the Mann–Whitney *U* test or the Kruskal–Wallis test (followed by Dunn–Bonferroni post hoc test). All reported *p* values were adjusted for multiple comparisons. We adopted the Chi-square test for categorical variables. Differences were considered statistically significant at* p* < 0.05.

For the present study, no authorization to an Ethics Committee was asked, because the original reports, nor this work, provided any personal information of the patients.

## Results

Our literature search identified 101 papers, including 37 case reports, 12 case series, 3 reviews with case reports, 42 reviews, 4 letters, 1 original article, 1 point of view, and 1 brief report. Four and one patients were excluded from the analysis because of a missing laboratory-proven SARS-CoV-2 infection or an ambiguous GBS diagnosis [disease course resembling chronic inflammatory demyelinating neuropathy (CIDP)], respectively. A total of 52 studies were included in the final analysis (total patients = 73) [5–56]. All data concerning the analyzed patients are reported in Table 1. For one case [20], most clinical and diagnostic details were not reported; therefore, many of our analyses were limited to 72 patients.

### Epidemiological distribution and demographic characteristics of the patients

**Fig. 1:**
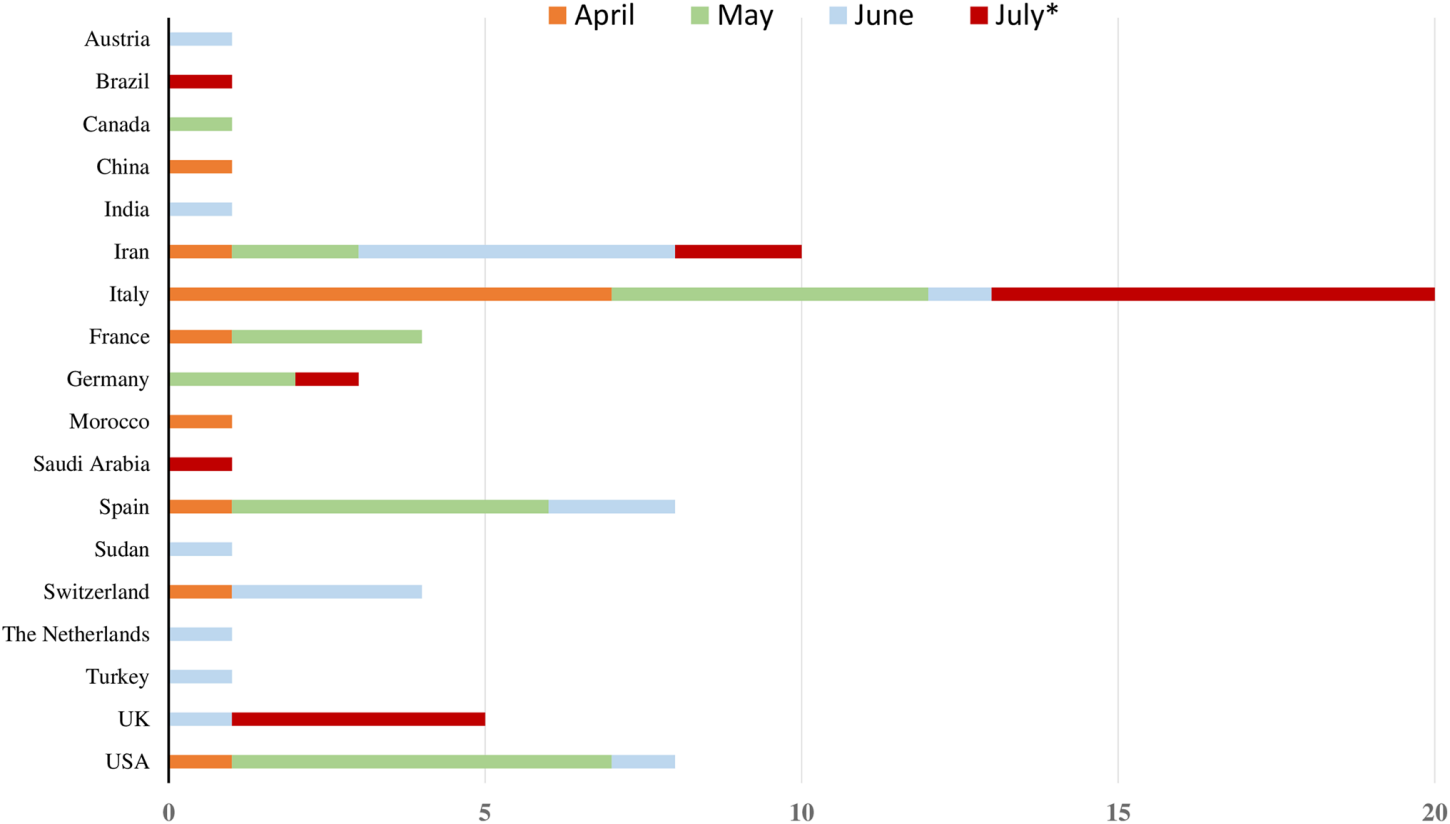
Temporal and spatial distribution of reported cases with COVID-19-associated Guillain–Barré syndrome in literature from 1st January until 20th July 2020. The *x*-axis shows the number of described patients. The *y*-axis illustrates the countries of provenience of the cases. In each line, different colours represent the months of April, May, June, and July (* until 20th July) 2020, in which the cases were published. Abbreviations: UK, United Kingdom, USA, United States of America

To date, GBS cases (*n* = 73) were reported from all continents except Australia. In details, patients were originally from Italy (*n* = 20), Iran (*n* = 10), Spain (*n* = 9), USA (*n* = 8), United Kingdom (*n* = 5), France (*n* = 4), Switzerland (*n* = 4), Germany (*n* = 3), Austria (*n* = 1), Brazil (*n* = 1), Canada (*n* = 1), China (*n* = 1), India (*n* = 1), Morocco (*n* = 1), Saudi Arabia (*n* = 1), Sudan (*n* = 1), The Netherlands (*n* = 1), and Turkey (*n* = 1) (Table 1, Fig. 1). The mean age at onset was 55 ± 17 years (min 11–max 94), including four pediatric cases [21, 27, 35, 41]. A significative prevalence of men compared to women was noticed (50 vs. 23 cases: 68.5% vs. 31.5%) with no significant difference in age at onset between men and women (mean: 55 ± 18 vs. 56 ± 16 years, *p* = 0.643). Comorbidities were variably reported with no prevalence of a particular disease.

### Clinical picture, diagnosis, and therapy of COVID-19

All reported GBS cases (*n* = 72) except two were symptomatic for COVID-19 with various severity. Most common manifestations of COVID-19 included fever (73.6%, 53/72), cough (72.2%, 52/72), dyspnea and/or pneumonia (63.8%, 46/72), hypo-/ageusia (22.2%, 16/72), hypo-/anosmia (20.8%, 15/72), and diarrhea (18.1%, 13/72). One of the two asymptomatic subjects never developed fever, respiratory symptoms, or pneumonia [10], whereas the other patient showed an asymptomatic pneumonia at chest computed tomography (CT) [12]. In all but six patients with available data [22, 24, 36, 44, 45, 52], SARS-CoV-2 RT-PCR with naso- or oropharyngeal swab or fecal exam was positive at first or following tests. Nevertheless, these six patients tested positive at SARS-CoV-2 serology. In four patients, the laboratory exam for the diagnostic confirmation was not specified [20, 40]. Typical “ground glass” aspects at chest-CT or similar findings at CT, Magnetic Resonance Imaging (MRI) or X-ray compatible with COVID-19 interstitial pneumonia were reported in 40 cases. The detailed therapies for COVID-19 are described in Table 1.

### Clinical features of GBS spectrum

In all (*n* = 72) but four patients [10, 37, 40, 56], GBS manifestations developed after those of COVID-19 [median (IQR): 14 (7–20), min 2–max 33 days]. Differently, COVID-19 symptoms began concurrent in one case [37], 1 day [40] and 8 days [55] after GBS onset in two other cases and never developed in another one [10] (Table 1). Common clinical manifestations at onset included sensory symptoms (72.2%, 52/72) alone or in combination with paraparesis or tetraparesis (65.2%, 47/72, respectively). Cranial nerve involvement (e.g., facial, oculomotor nerves) was less frequently described at onset (16.7%, 12/72). Moreover, all cases but one [26] showed lower limbs or generalized areflexia, whereas in 37.5% (27/72) of the cases, gait ataxia was reported at onset or during the disease course. Even if ascending weakness evolving into flaccid tetraparesis (76.4%, 55/72) and spreading/persistence of sensory symptoms (84.7%, 61/72) represented the most common clinical evolutions, 50.0% (36/72) and 23.6% (17/72) patients showed cranial nerve deficits and dysphagia, respectively, during disease course (Table 1). Moreover, 36.1% (26/72) of the patients developed respiratory symptoms, and some of them evolved to respiratory failure (Table 1). Autonomic disturbances were rarely reported (16.7%, 12/72). In cases with MFS/MFS-GBS overlap, areflexia, oculomotor disturbances, and ataxia were present in 100% (9/9), 66.7% (6/9) and 66.7% (6/9), respectively [8, 19, 23, 30, 32, 33, 43, 44]. The median of time to nadir was calculated in 40 patients with available data and resulted 4 days (IQR 3–9) (Table 1).

### Results of electrophysiological, CSF, biochemical, and neuroimaging investigations

Detailed electroneurography results were reported in 84.9% (62/73) of the cases. Specifically, 77.4% (48/62) cases showed a pattern compatible with a demyelinating polyradiculoneuropathy. In contrast, axonal damage was prominent in 14.5% (9/62). In a minority of the patients (8.1%), a mixed pattern was reported (5/62). Regarding CSF analysis (full results were available in 59 out of 73 cases), the classical albuminocytological dissociation (cell count < 5/µl with elevated CSF proteins) was detected in 71.2% of the cases (42/59) with a median CSF protein of 100.0 mg/dl (min: 49, max: 317 mg/dl). Mild pleocytosis (i.e., cell count ≥ 5/µl), with a maximum cell count of 13/µl, was evident in 5/59 cases (8.5%). Furthermore, CSF SARS-CoV-2 RNA was undetectable in all tested patients (*n* = 31) (Table 1).

Detailed blood haematological and biochemical examinations showed variably leucocytosis (*n* = 4), leucopenia (*n* = 17), thrombocytosis (*n* = 3), thrombocytopenia (*n* = 5), and increased levels of C-reactive protein (CRP) (*n* = 22), erythrocyte sedimentation rate (*n* = 4), d-Dimer (*n* = 5), fibrinogen (*n* = 3), ferritin (*n* = 3), LDH (*n* = 7), IL-6 (*n* = 4), IL-1 (*n* = 3), IL-8 (*n* = 3), and TNF-α (*n* = 3) (Table 1).

Furthermore, anti-GD1b and anti-GM1 antibodies were positive in one patient with MFS [23] and in one with classic sensorimotor GBS [13], respectively, whereas 33 cases tested negative (one in equivocal range) for anti-ganglioside antibodies.

Cranial and spinal MRI scans were performed in a minority of the patients (23/73, 31.5%). Five patients (three cases with AIDP [9, 12, 25], one case with MFS [30], and one case with bilateral facial palsy with paresthesia [52]) showed cranial nerve contrast enhancement in the context of correspondent cranial nerve palsies. Moreover, brainstem leptomeningeal enhancement was described in two cases with AIDP, both with clinical cranial nerve involvement [18, 46]. On the other hand, spinal nerve roots and leptomeningeal enhancement were reported in eight [9, 27, 31, 36, 37, 42, 52] and two cases [17, 46], respectively (Table 1).

### Distribution of clinical and electrophysiological variants and diagnosis of GBS

From the clinical point of view, most examined patients presented with a classic sensorimotor variant (70.0%, 51/73), whereas Miller Fisher syndrome, GBS/MFS overlap variants (including polyneuritis cranialis), bilateral facial palsy with paresthesia, pure motor, and paraparetic were described in seven, two, five, four, and one patients, respectively. In three cases, no clinical variant could be established using the reported details (Table 1). In the examined population, 81.8% subjects fulfilled electrophysiological criteria for AIDP (45/55), 12.7% (7/55) for AMSAN, and 5.4% (3/55) for AMAN subtypes. Finally, a specific electrophysiological subtype was not attributable in 18 patients due to the lack of detailed information. The diagnosis of GBS was established based on clinical, CSF, and electrophysiological findings in 44/73 (60.3%) patients, clinical, and electrophysiological data in 18/73 (24.7%) cases, clinical, and CSF data in 8/73 (11.0%), and only clinical findings in 3/73 (4.1%) patients. Indeed, the highest level of diagnostic certainty (level one) was confirmed in 44/73 cases (60.3%). Level two and three were obtained in 24/73 cases (32.9%) and 5/73 (6.8%), respectively (Table 1).

### Management of GBS and patient outcomes

All cases with available therapy data (*n* = 70) except ten [13, 15, 23, 25, 26, 33, 35–37, 41] were treated with intravenous immunoglobulin (IVIG) (Table 1). Conversely, plasma exchange and steroid therapy were performed in ten (four of them received also IVIG) and two cases, respectively. In two patients, no therapy was given. Mechanical or non-invasive ventilation was implemented in 21.4% (15/70) and 7.1% (5/70) patients due to worsening of GBS or COVID-19, respectively. At further observation (n = 68), 72.1% (49/68) patients demonstrated clinical improvement with partial or complete remission, 10.3% (7/68) cases showed no improvement, 11.8% (8/68) still required critical care treatment, and 5.8% (4/68) died (Table 1).

**Table 1:** Summary of clinical findings, results of diagnostic investigations, and outcome in 73 GBS cases

Article	Country	Age	Sex	GBS clinical picture						COVID-19 clinical picture	Previous comorbidities	GBS diagnosis	Level of diagnostic certainty^b^	GBS variant
				Days between COVID-19 symptoms and GBS onset	Onset	Disease course	Autonomic disturbances	Respiratory symptoms/failure	Time to Nadir^a^					
Agosti et al. [5]	Italy	68	M	5 days after	LL weakness	Bilateral facial palsy, progressive symmetric ascending flaccid tetraparesis, achilles tendon areflexia	NA	No	NA	Dry cough associated with fever, dysgeusia, and hyposmia	Dyslipidemia, benign prostatic hypertrophy, hypertension, abdominal aortic aneurysm	Clinical + CSF + electrophysiology	1	Pure motor
Alberti et al. [6]	Italy	71	M	4 days after (no resolution of pneumonia)	LL paraesthesia	Ascendant weakness, flaccid tetraparesis, hypoesthesia and paraesthesia in the 4 limbs, generalized areflexia, dyspnea	None	Yes (concurrent pneumonia)	4 days after symptoms onset (24 h after the admission)	Fever (low grade), dyspnea, pneumonia	Hypertension, treated abdominal aortic aneurysm, treated lung cancer	Clinical + CSF + electrophysiology	1	Classic sensorimotor
Arnaud et al. [7]	France	64	M	23 days after	Fast progressive LL weakness	Generalized areflexia, severe flaccid proximal paraparesis, decreased proprioceptive length-dependent sensitivity and LL pinprick and light touch hypoesthesia	None	No	4 days after symptoms onset	Fever, cough, diarrhea, dyspnea, severe interstitial pneumonia	DM type 2	Clinical + CSF + electrophysiology	1	Classic sensorimotor
Assini et al. [8]	Italy	55	M	20 days after	Bilateral eyelid ptosis, dysphagia, dysphonia	Masseter weakness, tongue protusion (bilateral hypoglossal nerve paralysis), UL and LL hyporeflexia without muscle weakness, soft palate elevation defect	None	Yes (concurrent pneumonia)	NA	Fever, anosmia, ageusia, cough, pneumonia	NA	Clinical + electrophysiology	2	Classic sensorimotor overlapping with Miller-Fisher
Assini et al. [8]	Italy	60	M	20 days after	Distal tetraparesis with right foot drop, autonomic disturbances	UL and LL distal weakness, right foot drop, generalized areflexia	Gastroplegia, paralytic ileus, loss of blood pressure control	Yes (concurrent pneumonia)	NA	Fever, severe interstitial pneumonia	NA	Clinical + electrophysiology	2	Pure motor
Bigaut et al. [9]	France	43	M	21 days after	UL and LL paraesthesia, distal LL weakness	Extension to midthigh and tips of the finger with ataxia, right peripheral facial nerve palsy, generalized areflexia	None	No	2 days after symptoms onset	Cough, asthenia, myalgia in legs, followed by acute anosmia and ageusia with diarrhea, mild interstitial pneumonia	NA	Clinical + CSF + electrophysiology	1	Classic sensorimotor
Bigaut et al. [9]	France	70	F	10 days after	Acute proximal tetraparesis, distal forelimb and perioral paraesthesia	Respiratory weakness, loss of ambulation	None	Yes	3 days after symptoms onset	Anosmia, ageusia, diarrhea, asthenia, myalgia, moderate interstitial pneumonia	Obesity	Clinical + CSF + electrophysiology	1	Classic sensorimotor
Bracaglia et al. [10]	Italy	66	F	Unknown (due to asymptomatic infection)	Acute proximal and distal tetraparesis, lumbar pain and distal tingling sensation	Loss of ambulation, difficulty in speeching and swallowing, generalized areflexia	None	No	NA	Asymptomatic	None	Clinical + electrophysiology	2	Classic sensorimotor
Camdessanche et al. [11]	France	64	M	11 days after	UL and LL paraesthesia	Ascendent weakness, flaccid tetraparesis, generalized areflexia, dysphagia	None	Yes	3 days after symptoms onset	Fever (high grade), cough, pneumonia	None	Clinical + CSF + electrophysiology	1	Classic sensorimotor
Chan et al. [12]	Canada	58	M	20 days after home isolation for suspected contact	Bilateral facial weakness, dysarthria, feet paraesthesia, LL areflexia	NA	None	No	NA	Asymptomatic, interstitial pneumonia	None	Clinical + CSF + electrophysiology	1	Bilateral facial palsy with paraesthesia
Chan et al. [13]	USA	68	M	18 days after	Gait disturbance, hands and feet paraesthesia	LL proximal weakness, absent vibratory and proprioceptive sense at the toes, UL hyporeflexia, LL areflexia, unsteady gait with inability to toe or heel walk, bilateral facial weakness, dysphagia, dysarthria, neck flexion weakness	None	No	8 days after the onset of symptoms	Fever and upper respiratory symptoms	NA	Clinical + CSF	2	Classic sensorimotor
Chan et al. [13]	USA	84	M	16 days after	Hands and feet paraesthesia, progressive gait disturbance	Bilateral facial weakness, progressive arm weakness, neuromuscular respiratory failure	Yes (not specified autonomic dysfunction)	Yes	25 days after the onset of symptoms	Fever	NA	Clinical + CSF	2	Classic sensorimotor
Coen et al. [14]	Switzerland	70	M	6 days after	Paraparesis, distal allodynia	Generalized areflexia	Difficulties in voiding and constipation	No	NA	Dry cough, myalgia, fatigue	None	Clinical + CSF + 0electrophysiology	1	Classic sensorimotor
Ebrahimzadeh et al. [15]	Iran	46	M	18 days after	Pain and numbness in distal LL and UL extremities, ascending weakness in legs	Mild peripheral right facial nerve palsy, generalized areflexia	None	No	7 days after symptoms onset	Low-grade fever, sore thorat, dry cough and mild dyspnea, bilateral interstitial pneumonia (concurrent with neurological symptoms)	None	Clinical + CSF + electrophysiology	1	Classic sensorimotor
Ebrahimzadeh et al. [15]	Iran	65	M	10 days after	Progressive ascending LL and UL extremities weakness and paraesthesia	Proximal and distal UL and LL weakness, UL hyporeflexia and LL areflexia	None	No	14 days after symptoms onset	History of COVID-19 (symptoms not specified), fine crackles in both lungs (concurrent with neurological symptoms)	Hypertension	Clinical + electrophysiology	2	Classic sensorimotor
El Otmani et al. [16]	Morocco	70	F	3 days after	Weakness and paraesthesia in the 4 limbs	Tetraparesis, hypotonia, generalized areflexia, bilateral positive Lasègue sign	None	No	NA	Dry cough, pneumonia	Rheumatoid arthritis	Clinical + CSF + electrophysiology	1	Classic sensorimotor
Esteban Molina et al. [17]	Spain	55	F	14 days after	Paraesthesia and weakness in the 4 limbs	Lumbar pain, dysphagia, tetraplegia, general areflexia, bilateral facial palsy, lingual and perioral paraesthesia	None	Yes	3 days after symptoms onset (48 h after the admission)	Fever, dry cough and dyspnoea, pneumonia	Dyslipidemia	Clinical + CSF + electrophysiology	1	Classic sensorimotor
Farzi et al. [18]	Iran	41	M	10 days after	Paraesthesia of the feet	Tetraparesis, areflexia at the LL and hyporeflexia at the UL, stocking-and-glove hypesthesia and reduced sense of vibration and position	None	No	7 days after symptoms onset	Cough, dyspnea and fever	DM type II	Clinical + electrophysiology	2	Classic sensorimotor
Fernández–Domínguez et al. [19]	Spain	74	F	15 days after	Gait ataxia and generalized areflexia	NA	NA	No	NA	Respiratory symptoms (not further detailed)	Hypertension and follicular lymphoma	Clinical + CSF	2	Miller Fisher variant
Finsterer et al. [20]	India	20	M	5 days after	NA	NA	NA	NA	NA	NA	NA	Clinical + electrophysiology	2	NA
Frank et al. [21]	Brazil	15	M	> 5 days after	Paraparesis, pain in the LL	Rapidly progressive ascending tetraparesis, areflexia	NA	No	NA	Fever, intense sweating	NA	Clinical + electrophysiology	2	Classic sensorimotor
Gigli et al. [22]	Italy	53	M	NA	Paraesthesia, gait ataxia	NA	NA	NA	NA	Fever, diarrhea	NA	Clinical + CSF + electrophysiology	1	NA
Gutiérrez-Ortiz et al. [23]	Spain	50	M	3 days after	Vertical diplopia, perioral paraesthesia, gait ataxia	Right internuclear ophthalmoparesis and right fascicular oculomotor palsy, ataxia, generalized areflexia	None	No	NA	Fever, cough, malaise, headache, low back pain, anosmia, ageusia	Bronchial asthma	Clinical + CSF	2	Miller Fisher variant
Gutiérrez-Ortiz et al. [23]	Spain	39	M	3 days after	Diplopia (bilateral abducens palsy)	Generalized areflexia	None	No	NA	Diarrhea, low-grade fever	None	Clinical + CSF	2	Polyneuritis cranialis (GBS–Miller Fisher Interface)
Helbok et al. [24]	Austria	68	M	14 days after	Hypoaesthesia and paraesthesia in the LL, proximal weakness, areflexia, stand ataxia	Ascending weakness, flaccid tetraparesis, generalized areflexia	NA	Yes	2 days after symptoms onset (24 h after the admission)	Fever, dry cough, myalgia, anosmia and ageusia.	None	Clinical + CSF + electrophysiology	1	Classic sensorimotor
Hutchins et al. [25]	USA	21	M	16 days after	Right-sided facial numbness and weakness	Bilateral facial palsy, severe dysarthria, bilateral LL weakness , bilateral UL paraesthesia, areflexia	NA	No	3 days after symptoms onset	Fever, cough, dyspnoea, diarrhea, nausea, headache	Hypertension, prediabetes, and class I obesity	Clinical + CSF + electrophysiology	1	Bilateral facial palsy with paraesthesia
Juliao Caamaño et al. [26]	Spain	61	M	10 days after	Facial diplegia	No progression	None	No	1 day after symptoms onset	Fever and cough	None	Clinical + electrophysiology	3	Bilateral facial nerve palsy
Khalifa et al. [27]	Kingdom of Saudi Arabia	11	M	20 days after	Gait ataxia, areflexia and paraesthesia in the LL	Gradual motor improvement, persistent hyporeflexia	NA	No	NA	Acute upper respiratory tract infection, low-grade fever, dry cough.	NA	Clinical + CSF + electrophysiology	1	Classic sensorimotor
Kilinc et al. [28]	The Netherlands	50	M	24 days after	Facial diplegia, symmetrical proximal weakness, paraesthesia of distal extremities, gait ataxia, areflexia	Progression of limb weakness and inability to walk	NA	No	11 days after symptoms onset	Dry cough	None	Clinical + electrophysiology	2	Classic sensorimotor
Lampe et al. [29]	Germany	65	M	2 days after	Acute right UL and LL weakness causing recurrent falls	Right UL paresis, slight paraparesis more pronounced on the right side, generalized hyporeflexia	None	No	3 days after symptoms onset	Fever and dry cough	None	Clinical + CSF + electrophysiology	1	Pure motor
Lantos et al. [30]	USA	36	M	4 days after	Opthalmoparesisa and hypoesthesia below knee	Progressive ophthalmoparesis (including initial left III cranial nerve and eventual bilateral VI cranial nerve palsies), ataxia, and hyporeflexia	None	No	NA	Fever, chills, and myalgia	None	Clinical	3	Miller Fisher variant
Lascano et al. [31]	Switzerland	52	F	15 days after (no resolution of pneumonia)	Back pain, diarrhea, rapidly progressive tetraparesis, distal paraesthesia	Worsening of proximal weakness (tetraplegia), generalized areflexia, ataxia	Constipation, abdominal pain	Yes	4 days after symptoms onset	Dry cough, dysgeusia, cacosmia	None	Clinical + CSF + electrophysiology	1	Classic sensorimotor
Lascano et al. [31]	Switzerland	63	F	7 days after (no resolution of pneumonia)	Limb weakness, pain on the left calf	Moderate tetraparesis, LL and left UL areflexia, distal hypoesthesia and paraesthesia	None	No	5 days after symptoms onset	Dry cough, shivering, breathing difficulties, chest pain, odynophagia	DM type 2	Clinical + electrophysiology	2	Classic sensorimotor
Lascano et al. [31]	Switzerland	61	F	22 days after	LL weakness, dizziness, dysphagia	Moderate tetraparesis, bilateral facial palsy, lower limb allodynia, severe hypopallesthesia, areflexia (except for bicipital tendon reflexes)	None	Yes	4 days after symptoms onset	Productive cough, headaches, fever, myalgia, diarrhea, nausea, vomiting, weight loss, recurrent episodes of transient loss of consciousness	None	Clinical + CSF + electrophysiology	1	Classic sensorimotor
Manganotti et al. [32]	Italy	50	F	16 days after	Diplopia and facial paraesthesia	Ataxia, diplopia in vertical and lateral gaze, left upper arm dysmetria, generalized areflexia, mild lower facial defects, and mild hypoesthesia in the left mandibular and maxillary branch	None	Yes (concurrent pneumonia)	NA	Fever, cough, ageusia, bilateral pneumonia	None	Clinical + CSF	2	Miller Fisher variant
Manganotti et al. [33]	Italy	72	M	18 days after	Tetraparesis UL > LL, LL paraesthesia , generalized areflexia, facial weakness on the right side	NA	NA	No	NA	Fever, dyspnea, hyposmia and ageusia	NA	Clinical + CSF + electrophysiology	1	Classic sensorimotor
Manganotti et al. [33]	Italy	72	M	30 days after	Tetraparesis LL > UL, paraesthesia, global areflexia	NA	NA	No	NA	Fever, cough, dyspnea, hyposmia and ageusia	NA	Clinical + electrophysiology	1	Classic sensorimotor
Manganotti et al. [33]	Italy	49	F	14 days after	Ophthalmoplegia, limb ataxia, generalized areflexia, diplopia, facial hypoesthesia, facial weakness	NA	NA	No	NA	Fever, cough, dyspnea, hyposmia and ageusia	NA	Clinical + CSF + electrophysiology	1	Miller Fisher variant
Manganotti et al. [33]	Italy	94	M	33 days after	LL weakness, generalized hyporeflexia	NA	NA	No	NA	Fever, cough, gastrointestinal symptoms	NA	Clinical + electrophysiology	2	Classic sensorimotor
Manganotti et al. [33]	Italy	76	M	22 days after	Quadriparesis UL > LL, generalized areflexia, facial weakness, transient diplopia	NA	NA	No	NA	Fever, cough, dysuria, hyposmia, ageusia	NA	Clinical + CSF + electrophysiology	1	Pure motor
Marta-Enguita et al. [34]	Spain	76	F	8 days after	Back pain and progressive tetraparesis with distal-onset paraesthesia	Progressive with dysphagia and cranial nerves involvement, generalized areflexia	NA	Yes	10 days after symptom onset	Cough and fever without dyspnea	None	Clinical	3	NA
Mozhdehipanah et al. [35]	Iran	38	M	16 days after	Progressive LL paraesthesia, facial diplegia, lobal areflexia	Mild LL weakness , bulbar symptoms developed	Blood pressure instability, tachycardia	No	8 days after symptoms onset	Upper respiratory infection (no further details)	NA	Clinical + CSF + electrophysiology	1	Bilateral facial palsy with paraesthesia
Mozhdehipanah et al. [35]	Iran	14	F	NA	Ascending quadriparesis, UL hyporeflexia, LL areflexia, distal hypoesthesia, ataxia	NA	NA	No	NA	Upper respiratory infection (no further details)	NA	Clinical + CSF	2	Classic sensorimotor
Mozhdehipanah et al. [35]	Iran	44	F	26 days after	Weakness of LL	Tetraparesis, generalized areflexia, symmetrical hypoesthesia	NA	Yes	NA	Dry cough, fever, myalgia, progressive dyspnea	COPD	Clinical + CSF + electrophysiology	1	Classic sensorimotor
Mozhdehipanah et al. [35]	Iran	66	F	30 days after	Progressive UL and LL weakness, generalized areflexia, symmetrical hypoesthesia	NA	No	No	NA	Fever, dry cough, severe myalgia	DM, hypertension, and rheumatoid arthritis	Clinical + CSF + electrophysiology	1	Classic sensorimotor
Naddaf et al. [36]	USA	58	F	17 days after	Progressive paraparesis, imbalance, severe lower thoracic pain without radiation	Mild neck flexion weakness, mild/moderate distal UL and proximal and distal LL weakness, UL hyporeflexia, LL areflexia, moderately severe length-dependent sensory loss in the feet, ataxic gait	None	No	NA	Fever, dysgeusia without anosmia, bilateral interstitial pneumonia	None	Clinical + CSF + electrophysiology	1	Classic sensorimotor
Oguz-Akarsu et al. [37]	Turkey	53	F	Concurrent pneumonia	Dysarthria, progressive LL weakness and numbness	Ataxia, generalized areflexia	None	No	NA	Mild fever (37.5 °C), pneumonia	None	Clinical + electrophysiology	2	Classic sensorimotor
Ottaviani et al. [38]	Italy	66	F	7 days after (concurrent pneumonia)	Flaccid paraparesis, no sensory symptoms	Progressively developed proximal weakness in all limbs, dysesthesia, and unilateral facial palsy, generalized areflexia	NA	Yes	13 days after symptoms onset	Fever and cough, pneumonia	NA	Clinical + CSF + electrophysiology	1	Classic sensorimotor
Padroni et al. [39]	Italy	70	F	23 days after	UL and LL paraesthesia, gait difficulties, asthenia	Ascendant weakness, tetraparesis, generalized areflexia	None	Yes	6 days after symptoms onset	Fever (38.5 °C), dry cough, pneumonia	None	Clinical + CSF + Electrophysiology	1	Classic sensorimotor
Paterson et al. [40]	UK	42	M	13 day after	Distal limb numbness and weakness, dysphagia	Tetraparesis, generalized areflexia, sensory loss	NA	Yes	16 days after symptom onset	Cough, fever dyspnea, diarrhea, anosmia	None	Clinical + CSF + electrophysiology	1	Classic sensorimotor
Paterson et al. [40]	UK	60	M	1 day before	Distal limb numbness and weakness	Tetraparesis, generalized areflexia, sensory loss, dysautonomia, facial and bulbar weakness	Yes	Yes	5 days after symptom onset	Headache, ageusia, anosmia	NA	Clinical + CSF + electrophysiology	1	Classic sensorimotor
Paterson et al. [40]	UK	38	M	21 day after	Distal limb numbness, weakness, clumsiness	Mild distal weakness, sensory ataxia	None	No	NA	Cough, diarrhea	NA	Clinical + CSF + electrophysiology	1	Classic sensorimotor
Paybast et al. [41]	Iran	38	M	21 days after	Acute progressive ascending paraesthesia of distal LL	Quadriparesthesia, bilateral facial droop with drooling of saliva and slurred speech, generalized areflexia, swallowing inability, bilaterally absent gag reflex	Tachycardia and blood pressure instability	No	3 days after symptoms onset	Symptoms of upper respiratory tract infection	Hypertension	Clinical + CSF + electrophysiology	1	Classic sensorimotor
Paybast et al. [41]	Iran	14	F	21 days after	Progressive ascending quadriparesthesia, mild LL weakness	Mild proximal and distal LL weakness, hypoactive deep tendon reflexes in UL and absent in LL, decreased light touch, position, and vibration sensation in all distal limbs up to ankle and elbow joints, gait ataxia	None	No	2 days after symptoms onset	Symptoms of upper respiratory tract infection	None	Clinical + CSF	2	Classic sensorimotor
Pfefferkorn et al. [42]	Germany	51	M	14 days after	UL and LL weakness, acral paraesthesia	Tetraparesis, generalized areflexia, deterioration to an almost complete peripheral locked-in syndrome with tetraplegia, complete sensory loss at 4 limbs, bilateral facial and hypoglossal paresis	None	Yes	15 days after symptoms onset	Fluctuating fever, flu-like symptoms with marked fatigue and dry cough, pneumonia	NA	Clinical + CSF + electrophysiology	1	Classic sensorimotor
Rana et al. [43]	USA	54	M	14 days after	LL paresthesias of LL	Ascending tetraparesis, general areflexia, burning sensation diplopia, facial diplegia, mild ophthalmoparesis	Resting tachycardia and urinary retention	Yes	NA	Rhinorrhea, odynophagia, fever, chills, and night sweats	Hypertension, hyperlipidemia, restless leg syndrome, and chronic back pain, concurrent *C. Difficile* infection	Clinical + electrophysiology	2	Miller Fisher variant
Reyes-Bueno et al. [44]	Spain	50	F	15 days after	Root-type pain in all four limbs, dorsal and lumbar back pain	LL Weakness, ataxia, diplopia, bilateral facial palsy, generalized areflexia	Dry mouth, diarrhea and unstable blood pressure	No	12 days after symptoms onset	Diarrhea, odynophagia and cough	NA	Clinical + CSF + electrophysiology	1	Miller Fisher variant
Riva et al. [45]	Italy	60+	M	17 days after	Progressive limb weakness and distal paresthesia at four limbs	Ascending paraparesis with involvement of the cranial nerves (facial diplegia), generalized areflexia	None	No	10 days after symptoms onset	Fever, headache, myalgia, anosmia and ageusia	NA	Clinical + electrophysiology	2	Classic sensorimotor
Sancho-Saldaña et al. [46]	Spain	56	F	15 days after	Unsteadiness and paraesthesia in both hands	Lumbar pain and ascending weakness, global areflexia, bilateral facial nerve palsy, oropharyngeal weakness and severe proximal tetraparesis	No	Yes	3 days after symptoms onset	Fever, dry cough and dyspnea, pneumonia	NA	Clinical + CSF + electrophysiology	1	Classic sensorimotor
Scheidl et al. [47]	Germany	54	F	11 days after	Proximal weakness of LL, numbness of 4 limbs	Initial worsening of the paraparesis with rapid improvement upon initiation of the treatment, areflexia	None	No	12 days after symptoms onset	Temporary ageusia,	None	Clinical + CSF + electrophysiology	1	Paraparetic variant
Sedaghat et al. [48]	Iran	65	M	14 days after	LL distal weakness	Ascending weakness, tetraparesis, facial bilateral palsy, generalized areflexia, LL distal hypoesthesia and hypopallesthesia	None	No	4 days after symptoms onset	Fever, cough and sometimes dyspnea, pneumonia	DM type 2	Clinical + electrophysiology	2	Classic sensorimotor
Sidig et al. [49]	Sudan	65	M	5 days after	Numbness and weakness in both UL and LL	Ascending weakness, bilateral facial paraesthesia and palsy, clumsiness of UL, tetraparesis, slight palatal muscle weakness, areflexia	Urinary incontinence	Yes	NA	Low-grade fever, sore throat, dry cough, headache and generalized fatigability	DM and Hypertension	Clinical + electrophysiology	2	Classic sensorimotor
Su et al. [50]	USA	72	M	6 days after	Proximal UL and LL weakness	Progression with worsening of the paresis, areflexia, hypoesthesia	Hypotension alternating with hypertension and tachycardia	Yes	8 days after symptoms onset	Mild diarrhea, anorexia and chills without fever or respiratory symptoms	Coronary artery disease, hypertension and alcohol abuse	Clinical + CSF + electrophysiology	1	Classic sensorimotor
Tiet et al. [51]	United Kingdom	49	M	21 days after	Distal LL paraesthesia	LL and UL weakness, facial diplegia, distal reduced sensation to pinprick and vibration sense, LL dysesthesia, generalized areflexia	None	No	4 days after symptoms onset	Shortness of breath, headache and cough	Sinusitis	Clinical + CSF + electrophysiology	1	Classic sensorimotor
Toscano et al. [52]	Italy	77	F	7 days after	UL and LL paraesthesia	Flaccid tetraplegia, areflexia, facial weakness, dysphagie, tongue weakness	None	Yes	NA	Fever, cough, ageusia, pneumonia	Previous ischemic stroke, diverticulosis, arterial hypertension, atrial fibrillation	Clinical + CSF + electrophysiology	1	Classic sensorimotor
Toscano et al. [52]	Italy	23	M	10 days after	Facial diplegia	LL paraesthesia, generalized areflexia, sensory ataxia	None	No	2 days after symptoms onset	Fever, pharyngitis	NA	Clinical + CSF + electrophysiology	1	Bilateral facial palsy with paraesthesia
Toscano et al. [52]	Italy	55	M	10 days after	Neck pain, Paresthesias in the 4 limbs, LL weakness	Flaccid tetraparesis, areflexia, facial weakness	None	Yes	NA	Fever, cough, pneumonia	NA	Clinical + CSF + electrophysiology	1	Classic sensorimotor
Toscano et al. [52]	Italy	76	M	5 days after	Lumbar pain, LL weakness	Flaccid tetraparesis, generalized areflexia, ataxia	None	No	4 days after symptoms onset	Cough and hyposmia	NA	Clinical + CSF+ Electrophysiology	1	Classic sensorimotor
Toscano et al. [52]	Italy	61	M	7 days after	LL weakness and paraesthesia	Ascending weakness, tetraplegia, facial weakness, areflexia, dysphagia	None	Yes	NA	Cough, ageusia and anosmia, pneumonia	NA	Clinical + CSF+ electrophysiology	1	Classic sensorimotor
Velayos Galán et al. [53]	Spain	43	M	10 days after	Distal weakness and numbness of the 4 limbs, gait ataxia	Progression of the weakness with bilateral facial paresis and dysphagia, generalized areflexia	NA	No	2 days after admission	Cough, pneumonia	NA	Clinical + electrophysiology	2	Classic sensorimotor
Virani et al. [54]	USA	54	M	8 days after	LL weakness, numbness	Ascending weakness, tetraparesis, areflexia	Urinary retention	Yes	Shortly after presentation in the outpatient clinic (after 2 days of symptoms onset)	Fever (102 F), dry cough, pneumonia	*Clostridium difficile* colitis 2 days before GBS onset	Clinical	3	Classic sensorimotor
Webb et al. [55]	United Kingdom	57		6 days after	Ataxia, progressive limb weakness and foot dysaesthesia,	Tetraparesis, generalized areflexia, hypoesthesia in the 4 limbs, hypopallesthesia in LL, dysphagia	None	Yes	3 days after symptoms onset	Mild cough and headache, myalgia and malaise, slight fever, diarrhea, pneumonia	Untreated hypertension and psoriasis	Clinical + CSF + electrophysiology	1	Classic sensorimotor
Zhao et al. [56]	China	61	F	8 days before	LL weakness	Ascending weakness, tetraparesis, areflexia, LL distal hypoesthesia	None	No	4 days after symptoms onset	Fever (38·2 °C), dry cough pneumonia	NA	Clinical + CSF + electrophysiology	1	Classic sensorimotor

Article	COVID-19 diagnosis	Blood findings	Auto-antibodies and screening for most common GBS causes	CSF findings	Electrophysiology: Neuropathy type and GBS electrophysiologic subtype	MRI (brain and spinal)	Management and therapy		Outcome
							GBS	COVID-19	
Agosti et al. [5]	RT-PCR + chest CT	Thrombocytopenia (101 × 10^9^ /L, reference value: 125–300 × 10^9^ /L), lymphocytopenia (0.48 × 10^9^ /L, reference value: 1.1–3.2 × 10^9^ /L)	Negative ANA, anti-DNA, c-ANCA, p-ANCA, negative screening for *Campylobacter jejuni*, *Mycoplasma pneumoniae*, *Salmonella enterica*, CMV, HSV 1 and 2, VZV, influenza virus A and B, HIV, normal B12 and serum protein electrophoresis	Increased total protein (98 mg/dl), cell count: 2/10^6^ L	Demyelinating AIDP	NA	IVIG 400 mg/kg/day (5 days)	Antiviral drugs (not specifically mentioned)	Improvement, discharged home after 30 days
Alberti et al. [6]	RT-PCR + chest CT	NA	NA	Increased total protein (54 mg/dl), 9 cells/µl, negative SARS-CoV-2 PCR	Demyelinating AIDP	NA	IVIG 400 mg/kg (5 days) + mechanical invasive ventilation	Lopinavir/ritonavir, hydroxychloroquine	24 h after admission, death because of respiratory failure
Arnaud et al. [7]	RT-PCR + chest CT	NA	Negative anti-ganglioside and antineural antibodies, negative *Campylobacter Jejuni*, HIV, syphilis, CMV, EBV serology	Increased total protein (1.65 g/L), no pleyocitosis, negative oligoclonal bands, negative SARS-CoV-2 PCR, negative EBV and CMV RT-PCR	Demyelinating AIDP	NA	IVIG 400 mg/kg (5 days)	Hydroxychloroquin, cefotaxime, azithromycine	Progressive improvement
Assini et al. [8]	RT-PCR	Lymphocytopenia, increased LDH and inflammation markers; low serum albumin (2.9 mg/dL)	NA	Normal total protein level, increased IgG/albumin ratio (233), negative SARS-CoV-2 PCR, presence of oligoclonal bands (both in serum and CSF)	Demyelinating with sural sparing AIDP	Brain: no pathological findings	IVIG 400 mg/kg (5 days)	Hydroxychloroquine, arbidol, ritonavir and lopinavir + mechanical invasive ventilation	5 days after IVIG, improvement of swallowing, speech, tongue motility, eyelid ptosis and strength
Assini et al. [8]	RT-PCR + chest CT	Lymphocytopenia, increased LDH and GGT, leucocytosis, low serum albumin (2.6 mg/dL)	Negative anti-ganglioside antibodies	Normal total protein level, increased IgG/albumin ratio (170), negative SARS-CoV-2 PCR, presence of oligoclonal bands (both in serum and CSF)	Motor sensory axonal, muscular neurogenic changes AMSAN	NA	IVIG 400 mg/kg (5 days)	Hydroxychloroquine, antiretroviral therapy, tocilizumab + tracheostomy and assisted ventilation	5 days after IVIG, improvement of vegetative symptoms, persistence of hyporeflexia and right foot drop
Bigaut et al. [9]	RT-PCR + chest CT	Normal blood count, negative CRP	Negative anti-ganglioside antibodies, negative HIV, Lyme and syphilis serology	Increased total protein (0.95 g/L), cell count: 1 × 10^6^/L, negative SARS-CoV-2 PCR	Demyelinating AIDP	Spinal: Radiculitis and plexitis on both brachial and lumbar plexus; multiple cranial neuritis (in III, VI, VII, and VIII nerves)	IVIG 400 mg/kg (5 days) + non-invasive ventilation	NA	Progressive improvement
Bigaut et al. [9]	RT-PCR + chest CT	Increased CRP	Negative anti-ganglioside antibodies	Increased total protein (1.6 g/L), cell count: 6 × 10^6^/L, negative SARS-CoV-2 PCR	Demyelinating AIDP	NA	IVIG 400 mg/kg (5 days)	NA	Slow progressive improvement
Bracaglia et al. [10]	RT-PCR (normal chest CT)	Elevated CPK (461 U/L, normal < 145), CRP 5,65 mg/dL (normal < 0.5), lymphocyto- penia (0·68 × 10^9^/L, normal 1·10^–4^), mild increase of LDH (284 U/L, normal < 248), GOT and GPT (549 and 547 U/L, normal < 35), elevation of IL-6 (11 pg/mL, normal < 5.9)	Negative anti-ganglioside antibodies; negative microbiologic testing on CSF and serum for HSV1-2, EBV, VZV, CMV, HIV, Mycoplasma Pneumoniae and Borrelia.	Increased total protein (245 mg/dL) and increased cell count: 13 cells/mm^3^, polymorphonucleate 61.5%	Demyelinating AIDP	NA	IVIG 400 mg/kg (5 days)	Hydroxychloroquine, ritonavir, darunavir	Improvement of UL and LL weakness, development of facial diplegia
Camdessanche et al. [11]	RT-PCR + chest CT	NA	Negative anti-gangliosides antibodies; negative screening for *Campylobacter jejuni, Mycoplasma pneumoniae*, Salmonella enterica, CMV, EBV, HSV1-2, VZV, Influenza virus A & B, HIV, and hepatitis E	Increased total protein (1.66 g/L), normal cell count	Demyelinating AIDP	NA	IVIG 400 mg/kg (5 days) + mechanical invasive ventilation	Oxygen therapy, paracetamol, low molecular weight heparin, lopinavir/ritonavir 400/100 mg twice a day for 10 days	NA
Chan et al. [12]	RT-PCR + chest CT	Persistent thrombocytosis (maximum PC 688 ×10^9^/L), elevated d-dimer (1.47 mg/L)	NA	Increased total protein (1.00 g/L), cell count: 4 × 10^6^/L (normal), negative SARS-CoV-2 PCR	Demyelinating AIDP	Brain: bilateral intracranial facial nerve enhancement	IVIG 400 mg/kg (5 days)	Empiric azithromycin and ceftriaxone	Slight improvement of facial weakness, unchanged paraesthesia
Chan et al. [13]	RT-PCR	NA	Negative anti-gangliosides antibodies	Increased total protein (226 mg/dL), leucocytes: 3 cells/mm^3^, glucose: 56 mg/dL, negative SARS-CoV-2 PCR	NA	Lumbosacral spine: no pathological findings	5 sessions of plasmapheresis	NA	Resolution of dysphagia, ambulation with minimal assistance 28 days after symptoms onset
Chan et al. [13]	RT-PCR	NA	Elevated GM2 IgG/IgM antibodies	Increased total protein (67 mg/dL), leucocytes: 1 cells/mm^3^, glucose 58 mg/dL, negative SARS-CoV-2 PCR	NA	NA	Mechanical invasive ventilation + 5 sessions of plasmapheresis (without benefit on ventilation) + IVIG	NA	Persistence of quadriparesis with intermittent autonomic dysfunction, slowly weaned from the ventilator
Coen et al. [14]	RT-PCR + serology	Normal (not specified)	Negative anti-gangliosides antibodies; negative meningitis/encephalitis panel	Albuminocytological dissociation, no intrathecal IgG synthesis, negative SARS-CoV-2 PCR	Demyelinating with sural sparing AIDP	Brain: NA Spinal: no pathological findings	IVIG 400 mg/kg (5 days)	NA	Rapid improvement. From day 11 from hospitalisation Rehabilitation
Ebrahimzadeh et al. [15]	RT-PCR + chest CT	Normal CRP (5 mg/L), normal serum protein immunoelectrophoresis	Negative anti-GQ1b antibodies, negative screening for *Campylobacter jejuni*, HIV, EBV, CMV, influenza virus (type A and B), HCV, non-reactive VDRL	Increased total protein (78 mg/dL), normal cell count (erythrocyte = 0/mm^3^, leukocyte = 4/mm^3^), normal glucose (70 mg/dL)	Demyelinating AIDP	Brain: no pathological findings Spinal: no pathological findings	None	Hydroxychloroquine for 5 days	Improvement of muscle strength to near normal after 16 days
Ebrahimzadeh et al. [15]	RT-PCR + chest CT	Slightly elevated CRP (34 mg/L), normal serum protein immunoelectrophoresis	Negative anti-GQ1b antibodies, negative screening for *Campylobacter jejuni*, HIV, EBV, CMV, influenza virus (type A and B), HCV, non-reactive VDRL	NA	Demyelinating AIDP	NA	IVIG	NA	Improvement of muscle strength in all extremities after 14 days
El Otmani et al. [16]	RT-PCR + chest CT	Lymphocytopenia (520/ml)	NA	Increased total protein (1 g/L), normal cell count, negative PCR assay for SARS-CoV-2	Motor sensory axonal AMSAN	NA	IVIG 400 mg/kg/day (5 days)	Hydroxychloroquine 600 mg/day; azithromycin 500 mg at the first day, then 250 mg per day	At week 1 from admission no significant neurological improvement
Esteban Molina et al. [17]	RT-PCR + chest X-ray	Leucocyte 7400/mm^3^, lymphocyte 2400/mm^3^. Hb 14 g/dl. PC 408,000/mm^3^, d-Dimer 556 ng/ml. Ferritin 544 ng/ml, CRP 2.04 mg/dl, Fibrinogen 6.8 g/dl	Negative bacteriological and viral tests	Increased total protein (86 mg/dL), cell count: 3x10^6^/L	Demyelinating AIDP	Brain: leptomeningeal enhancement in midbrain and cervical spine	IVIG 400 mg/kg/day (5 days)	Hydroxychloroquine, azithromycin, ceftriaxon	Motor improvement but persistence of paraesthesia
Farzi et al. [18]	RT-PCR + chest CT	Lymphopenia (WBC:5.9 × 10^9^/L, neutrophils: 85%, lymphocyte:15%), elevated levels of CRP, ESR 69 mm/h	NA	NA	Demyelinating AIDP	NA	IVIG (2 g/kg over 5 days)	Lopinavir/ritonavir and hydroxychloroquine	Improvement after 3 days, favorable outcome
Fernández–Domínguez et al. [19]	RT-PCR	NA	Negative anti-GD1b antibodies, negative other anti-ganglioside antibodies	Increased total protein (110 mg/dL), albuminocytological dissociation	Demyelinating NA	Brain: no pathological findings	IVIG 20 g/day (5 days)	Hydroxychloroquine, lopinavir/ritonavir	NA
Finsterer et al. [20]	NA	NA	NA	NA	Axonal AMAN	NA	IVIG	NA	Recovery
Frank et al. [21]	RT-PCR, + serology (IgG and IgM)	WBC and CRP normal	Negative hepatitis B and C, HIV and VDRL tests	Two CSF analysis 2 weeks apart, both showing normal cell count and CSF biochemistry, negative SARS-CoV-2 PCR, negative PCR for HSV1, HSV2, CMV, EBV, VZV; Zika virus; Dengue virus and Chikungunya virus	Axonal AMAN	Brain: no pathological findings Spinal: no pathological findings	IVIG 400 mg/kg/day (5 days)	Methylprednisolone, azithromycin, albendazole	Some improvement, weakness persisted
Gigli et al. [22]	Chest CT + serology (negative RT-PCR)	NA	Negative anti-ganglioside antibodies, negative PCR for influenza A and B viruses (nasal swab)	Increased total protein (192.8 mg/L), leucocytes: 2.6 cells/µL, positive Ig for SARS-CoV-2, negative SARS-CoV-2 PCR	Demyelinating AIDP	NA	NA	NA	NA
Gutiérrez-Ortiz et al. [23]	RT-PCR	Lymphocytes 1000 cells/UI, CRP 2.8 mg/dl	Positive anti-GD1b antibodies, other anti-ganglioside antibodies negative	Increased total protein (80 mg/dl), no leucocytes, glucose 62 mg/dl, negative SARS-CoV-2 PCR	NA	NA	IVIG 400 mg/kg (5 days)	NA	After 2 weeks from admission complete resolution except anosmia, ageusia
Gutiérrez-Ortiz et al. [23]	RT-PCR	Leucopenia (3100 cells/µl)	NA	Increased total protein (62 mg/dl), WBC: 2/μl (all monocytes), glucose: 50 mg/dl, negative SARS-CoV-2 PCR	NA	NA	None	Paracetamol	2 weeks later complete neurological recovery with no ageusia, complete eye movements, and normal deep tendon reflexes
Helbok et al. [24]	Chest CT + serology (repeated negative RT-PCR)	WBC 8.1G/L (normal: 4.0–10.0G/L), CRP 2.3 mg/dL, (normal: 0.0–0.5 mg/dL), fibrinogen level 650 mg/dL (normal: 210–400 mg/dL), LDH 276 U/L (normal: 100–250 U/L), erythrocyte sedimentation rate 55 mm/1 h	Negative PCR for CMV, EBV, influenza virus A/B, Respiratory Syncytial Virus and IgM antibodies for *Chlamydia pneumoniae* and *Mycoplasma pneumoniae*	Increased total protein (64 mg/dl), cell count: 2 cells/mm^3^, serum/ CSF glucose ratio of 0.83, negative SARS-CoV-2 PCR, positive anti-SARS-CoV-2 antibodies (not determined if intrathecal synthesis or passive transfer from blood)	Demyelinating with sural sparing AIDP	Spinal: no pathological findings	IVIG 30 g + plasma exchange (4 cycles) + mechanical invasive ventilation	None	Improvement of muscle forces with recovery of mobility without significant help after 8 weeks
Hutchins et al. [25]	RT-PCR + chest CT	Lymphopenia (absolute lymphocyte count of 0.7 K/mm^3^)	Serum HSV IgG and IgM. Respiratory viral panel PCR negative Negative GM1, GD1b, and GQ1b IgG and IgM), aquaporin-4 receptor (IgG), HIV 1/2, HSV 1/2 (IgG and IgM), CMV (IgM), *Mycoplasma pneumoniae* (IgG and IgM), *Borrelia burgdorferi* (IgG and IgM), *Bartonella* species (IgG and IgM), and syphilis (Venereal Disease Research Laboratory test)	Increased total protein (49 mg/dL), normal glucose levels (65 mg/dL), no leukocytes	Mixed demyelinating and axonal EMG subtype unknown	Brain: enhancement of the facial and abducens nerves bilaterally, as well as the right oculomotor nerve Spinal: no pathological findings	Plasma exchange (5 cycles)	NA	Discharged to inpatient rehabilitation
Juliao Caamaño et al. [26]	RT-PCR	NA	NA	Normal total protein (44 mg/dL), no pleocytosis	Absent blink-reflex EMG subtype unknown	Brain: no pathological findings	Oral prednisolone	Hydroxychloroquine and lopinavir/ritonavir for 14 days	Minimal improvement of muscle weakness after 2 weeks
Khalifa et al. [27]	RT-PCR + chest X-ray + chest CT	WBC 5.5 × 10^3^, PC 356 × 10^3^, CRP 0.5 mg/dL (normal 0.0–0.5), serum ferritin 87.3 ng/ml (normal 12.0–150.0), elevated d-Dimer levels 0.72 mg/L (0.00–0.49)	Negative screening for: influenza A and B viruses; influenza A virus subtypes H1, H3, and H5 including subtype H5N1 of the Asian lineage; parainfluenza virus types 1, 2, 3, and 4; respiratory syncytial virus types A and B; adenovirus; metapneumovirus; rhinovirus; enterovirus; Coronavirus 229E, HKU1, NL63, and OC43	Cell count: 5 mm^3^, increased total protein (316.7 mg/dL)	Demyelinating AIDP	Brain: no pathological findings Spinal: enhancement of the cauda equina nerve roots	IVIG 1 g/kg (2 days)	Paracetamol, azithromycin, hydroxychloroquine	Discharge to home after 15 days with clinical and electrophysiological improvement
Kilinc et al. [28]	Fecal PCR + serology	NA	Negative anti-GQ1b antibodies, serologic tests on *Borrelia burgdorferi*, syphilis, *Campylobacter jejuni*, CMV, hepatitis E, *Mycoplasma pneumoniae* and CMV	Normal cell count, normal proteins	Predominantly demyelinating AIDP	Brain: no pathological findings	IVIG 2 g/kg (5 days)	None	Persistence of mild symptoms at the discharge (after 14 days)
Lampe et al. [29]	RT-PCR (negative chest X-ray)	Slightly increased CRP (1.92 mg/dL)	Negative anti-ganglioside antibodies; negative influenza and respiratory syncytial virus	Increased total protein (56 mg/dL), normal cell count (2 cells/μL)	Demyelinating AIDP	NA	IVIG 400 mg/kg (5 days)	None	Improvement of GBS symptoms with persistence of generalized areflexia except for left biceps reflex, discharge after 12 days
Lantos et al. [30]	RT-PCR	NA	GM1 antibodies in the equivocal range	NA	NA	Brain: enlargement, prominent enhancement with gadolinium, and T2 hyperintense signal of the left cranial nerve III	IVIG	Hydroxychloroquine	Improvement, discharge after 4 days
Lascano et al. [31]	RT-PCR + chest X-ray + positive IgM (IgG positivity 2 weeks later)	WBC 8900 cells/mm^3^; lymphocytes 1200 cells/mm^3^; PC 45,500 cells/mm^3^	Negative anti-ganglioside antibodies	Increased total protein (60 mg/dL), leucocytes: 3 cells/μL, negative SARS-CoV-2 PCR	Demyelinating AIDP	Spinal: no nerve root gadolinium enhancement	IVIG 400 mg/kg (5 days) + mechanical invasive ventilation	Azithromycin	Improvement of tetraparesis. Able to stand up with assistance.
Lascano et al. [31]	RT-PCR + chest X-ray	WBC 3300 cells/mm^3^; lymphocytes 800 cells/mm^3^; PC 119,000 cells/mm^3^	NA	Normal total protein (40 mg/dl), cell count: 2 cells/μL	Mixed demyelinating (conduction blocks) and axonal with sural sparing pattern Predominantly AIDP	NA	IVIG 400 mg/kg (5 days)	Amoxicillin, clarithromycin	Dismissal with full motor recovery. Persistence of LL areflexia and distal paraesthesia
Lascano et al. [31]	RT-PCR + chest X-ray	WBC 4000 cells/mm^3^; lymphocytes 600 cells/mm^3^; PC 322,000 cells/mm^3^	NA	Increased total protein (140 mg/dL), cell count: 4 cells/μL, negative SARS-CoV-2 PCR	Demyelinating with sural sparing pattern AIDP	Brain: no pathological findings Spinal cord: lumbosacral nerve root enhancement	IVIG 400 mg/kg (5 days)	Amoxicillin	Improvement of tetraparesis and ability to walk with assistance. Persistence of neuropathic pain and distal paraesthesia
Manganotti et al. [32]	RT-PCR + chest CT	NA	Negative anti-ganglioside antibodies negative serum anti-HIV, anti-HBV, anti-HCV antibodies	Increased total protein (74.9 mg/dL), negative CSF PCR for bacteria, fungi, *Mycobacterium tuberculosis*, Herpes viruses, Enteroviruses, Japanese B virus and Dengue viruses	NA	Brain: no pathological findings	IVIG 400 mg/kg (5 days)	Lopinavir/ritonavir, hydroxychloroquine, antibiotic therapy, oxygen support (35%)	Resolution of all symptoms except for minor hyporeflexia at the LL
Manganotti et al. [33]	RT-PCR	IL-1: 0.2 pg/ml (< 0.001 pg/ml), IL-6: 113.0 pg/ml (0.8–6.4 pg/ml), IL-8: 20.0 pg/ml (6.7–16.2 pg/ml), TNF-α: 16.0 pg/ml (7.8–12.2 pg/ml)	Negative anti-ganglioside antibodies, negative HIV, HBV, HCV negative serological tests for autoimmune disorders	Increased total protein (52 mg/dl), leucocytes: 1 cell/mm^3^, negative SARS-CoV-2 PCR	Demyelinating AIDP	NA	IVIG 400 mg/kg/day (5 days)	Hydroxychloroquine, oseltamivir, darunavir, methylprednisolone and tocilizumab + mechanical invasive ventilation	Improvement of motor symptoms
Manganotti et al. [33]	RT-PCR	IL-1: 0.5 pg/ml (< 0.001 pg/ml), IL-6: 9.8 pg/ml (0.8–6.4 pg/ml), IL-8: 55.0 pg/ml (6.7–16.2 pg/ml), TNF- α: 16.0 pg/ml (7.8–12.2 pg/ml)	Negative anti-ganglioside antibodies, negative HIV, HBV, HCV negative serological tests for autoimmune disorders	Normal total protein (40 mg/dl), leucocytes: 1 cell/mm^3^, negative SARS-CoV-2 PCR	Mixed demyelinating and axonal EMG subtype unknown	Brain: no pathological findings	IVIG 400 mg/kg/day (5 days)	Hydroxychloroquine, lopinavir/ritonavir, methylprednisolone + mechanical invasive ventilation	Improvement of motor symptoms
Manganotti et al. [33]	RT-PCR	NA	Negative anti-ganglioside antibodies, negative HIV, HBV, HCV negative serological tests for autoimmune disordes	Increased total protein (72 mg/dL), leucocytes: 5 cell/mm^3^, negative SARS-CoV-2 PCR	Mainly demyelinating Predominantly AIDP	Brain: no pathological findings	IVIG 400 mg/kg/day (5 days)	Hydroxychloroquine, lopinavir/ritonavir, methylprednisolone	Improvement
Manganotti et al. [33]	RT-PCR	NA	NA	NA	Mixed demyelinating and axonal EMG subtype unknown	NA	Methylprednisolone 60 mg for 5 days	Methylprednisolone	Stationary
Manganotti et al. [33]	RT-PCR	IL-1: 0.2 pg/ml (< 0.001 pg/ml), IL-6: 32.7 pg/ml (0.8–6.4 pg/ml), IL-8: 17.8 pg/ml (6.7–16.2 pg/ml), TNF- α : 11.1 pg/ml (7.8–12.2 pg/ml), IL-2R: 1203.0 pg/ml (440.0–1435.0 pg/ml), IL-10: 4.6 (1.8–3.8 pg/ml)	Negative anti-ganglioside antibodies, negative HIV, HBV, HCV negative serological tests for autoimmune disordes	Increased total protein (53 mg/dL), leucocytes: 2 cell/mm^3^, negative SARS-CoV-2 PCR	Mixed demyelinating and axonal EMG subtype unknown	NA	IVIG 400 mg/kg/day (5 days)	Hydroxychloroquine, lopinavir/ritonavir, methylprednisolone, meropenem, linezolid, clarithromycin, fluconazole, doxycycline + mechanical invasive ventilation	Improvement
Marta-Enguita et al. [34]	RT-PCR + chest CT	Thrombocytopenia, d-Dimer elevation	NA	NA	NA	NA	NA	NA	Death after 10 days
Mozhdehipanah et al. [35]	RT-PCR (negative chest CT)	Normal WBC, CRP and ESR	NA	Increased total protein (139 mg/dL), normal cell count, negative CSF HSV serology and gram stain and culture	Demyelinating AIDP	NA	Plasma exchange (5 cycles)	NA	Significant improvement of muscle weakness after 3 weeks, persistence of mild bifacial paresis
Mozhdehipanah et al. [35]	RT-PCR	Normal WBC, CRP and ESR	NA	Albuminocytological dissociation	NA	NA	IVIG 400 mg/kg/day (5 days)	NA	Complete recovery, except for the persistence of hyporeflexia
Mozhdehipanah et al. [35]	RT-PCR + chest CT	Leucocytosis lymphopenia, elevated ESR and CRP	NA	Increased total protein (57 mg/dL), normal cell count and glucose (not further specified)	Axonal AMSAN	NA	IVIG 400 mg/kg/day (3 days)	Hydroxy chloroquine, lopinavir/ ritonavir	Death after 3 days from starting treatment with IVIG
Mozhdehipanah et al. [35]	RT-PCR + chest CT	Leucocytosis, lymphopenia, elevated ESR and CRP	NA	Increased total protein (89 mg/dL), normal cell count and glucose (not further specified)	Demyelinating AIDP	NA	IVIG 400 mg/kg/day (5 days)	Hydroxy chloroquine, lopinavir/ ritonavir	No significant clinical improvement
Naddaf et al. [36]	Positive SARS-CoV-2 IgG (index value: 8.2, normal < 0.8) and IgA + chest CT (negative RT-PCR)	Normal completed blood count, elevated d-dimer (690 ng/mL), ferritin (575 mcg/L), ESR (26 mm/h), alanine aminotransferase (73 U/L)	Negative anti-ganglioside antibodies negative HIV, syphilis, West Nile virus, Lyme disease testing, EBV and CMV serology consistent with remote infection, negative paraneoplastic evaluation	Increased total protein (273 mg/dL), total cells count: 2/mm3, negative CSF SARS-CoV-2 RT-PCR, negative meningitis/encephalitis panel, negative oligoclonal bands and IgG index	Demyelinating AIDP	Spine: smooth enhancement of the cauda equine roots	Plasma exchange (5 sessions)	Hydroxy chloroquine, zinc, methylprednisolone 40 mg bid for 5 days	Improvement of motor and gait examination. Persistence of slight ataxia without requiring gait aid
Oguz-Akarsu et al. [37]	RT-PCR + chest MRT + chest CT	Mild neutropenia (1.49 cells/µL) and a high monocyte percentage (19.77)	HIV test negative	Normal total protein (32.6 mg/dL) with no leucocytes	Demyelinating with sural sparing pattern AIDP	Cervical and lumbar and spine: asymmetrical thickening and hyperintensity of post-ganglionic roots supplying the brachial and lumbar plexuses in STIR sequences	Plasma exchange (five sessions, one every other day)	Hydroxychloroquine, azithromycin	Marked neurological improvement after 2 weeks and she was able to walk without assistance
Ottaviani et al. [38]	RT-PCR + chest CT	Lymphopenia, increased d-dimer, CRP and CK	Negative anti-ganglioside antibodies	Increased total protein (108 mg/dL), cell count: 0 cells/μL	Mainly demyelinating Predominantly AIDP	NA	IVIG 400 mg/kg (5 days)	Lopinavir/ritonavir, hydroxychloroquine	Progressive worsening with multi-organ failure
Padroni et al. [39]	RT-PCR + chest CT	WBC 10.41 × 10^9^/L (neutrophils 8.15 × 10^9^/L), normal d-dimer	Negative screening for *Mycoplasma pneumonia*, CMV, *Legionella pneumophila*, *Streptococcus pneumoniae*, HSV, VZV, EBV, HIV-1, *Borrelia burgdorferi*; auto-antibodies not performed	Increased total protein (48 mg/dl), cell count: 1 × 10^6^/L	Motor sensory axonal AMSAN	NA	IVIG 400 mg/kg (5 days) + mechanical invasive ventilation	NA	At day 6 from admission: ICU with mechanical invasive ventilation
Paterson et al. [40]	Definite diagnosis (not specified) (normal chest CT)	Increased neutrophils and CRP	NA	Increased total protein (0.5 g/L), leucocytes: 3 cells/μL (0–5),	Demyelinating AIDP	NA	IVIG + mechanical invasive ventilation	None	17 days of hospitalisation, at discharge able to walk 5 m (across an open space) but incapable of manual work/running
Paterson et al. [40]	Definite diagnosis (not specified) (normal chest CT)	Increased CRP and fibrinogen	NA	Increased total protein (0.6 g/L) leucocytes: 2 cells/μL (0-5), Glucose 3.4 (mmol/L; 2.2-4.2)	Demyelinating AIDP	Brain: no pathological findings	IVIG	Mechanical invasive ventilation	46 days (ongoing) of hospitalisation, still critical and requiring ventilation
Paterson et al. [40]	Definite diagnosis (not specified) (normal chest CT)	Not significant findings	NA	Increased total protein (0.9 g/L) leucocytes: < 1 cells/μL (0-5), Glucose 3.7 (mmol/L; 2.2-4.2)	Demyelinating AIDP	Brain: no pathological findings	IVIG	NA	7 days (ongoing) of hospitalisation, able to walk 5 m (across an open space) but incapable of manual work/running
Paybast et al. [41]	RT-PCR	NA	NA	Increased total protein (139 mg/dL), normal glucose and cell count, normal CSF viral serology, negative gram stain and culture	Mixed demyelinating and axonal EMG subtype unknown	NA	5 sessions of therapeutic plasma exchange, intravenous bolus of labetalol to control sympathetic nervous system over-reactivity	Hydroxychloroquine sulphate 200 mg two times per day for a week	Persistence of generalized hyporeflexia, decreased light touch sensation in distal limbs, mild bilateral facial paresis, sympathetic over-reactivity successfully controlled with labetalol,
Paybast et al. [41]	RT-PCR	NA	NA	Albuminocytological dissociation	NA	NA	IVIG 20 g (5 days)	Hydroxychloroquine sulphate 200 mg two times per day for a week	Persistence of generalized hyporeflexia and decreased light touch sensation in distal limbs
Pfefferkorn et al. [42]	RT-PCR + chest CT	NA	Negative anti-gangliosides antibodies	At admission: Normal total protein, cell count: 9/µL, negative SARS-CoV-2 PCR At day 13th: increased total protein (10.231 mg/L), normal cell count	Demyelinating AIDP	Spinal: massive symmetrical contrast enhancement of the spinal nerve roots at all levels of the spine including the cauda equina. Anterior and posterior nerve roots were equally affected	IVIG 30 g (5 days) + mechanical invasive ventilation + plasma exchange	NA	At day 31 from admission: motor improvement with regression of facial and hypoglossal paresis but still needed mechanical ventilation
Rana et al. [43]	RT-PCR	NA	NA	NA	Demyelinating with sural sparing AIDP	Thoracic and lumbar spine: no evidence of myelopathy or radiculopathy	IVIG 400 mg/kg (5 days)	Hydroxychloroquine and azithromycin	On day 4 respiratory improvement, on day 7 rehabilitation
Reyes-Bueno et al. [44]	Serology (negative RT-PCR)	NA	Negative anti-ganglioside antibodies	Increased total protein (70 mg/dl), cell count: 5 cells/µl, albuminocytological dissociation	Demyelinating with alteration of the Blink-Reflex. Further EMG: polyradiculoneuropathy with proximal and brainstem involvement AIDP	NA	IVIG 400 mg/kg (5 days) + Gabapentin	NA	After the 18th day progressive improvement of facial and limb paresis, diplopia and pain. Consequent neurological rehabilitation
Riva et al. [45]	Chest CT + serology (negative RT-PCR)	No pathological findings	Negative anti-ganglioside antibodies	Normal total protein and cells; negative PCR for SARS-CoV2, EBV, CMV, VZV, HSV 1–2, HIV	Demyelinating with sural sparing AIDP	Brain: NA Spinal: no pathological findings	IVIG 400 mg/kg (5 days)	None	Slowly improvement after the 10th day
Sancho-Saldaña et al. [46]	RT-PCR + chest X-Ray	NA	Negative anti-ganglioside antibodies	Increased total protein (0.86 g/L), cell count: 3 leucocytes	Demyelinating AIDP	Whole spine: brainstem and cervical meningeal enhancement	IVIG 400 mg/kg (5 days)	Hydroxychloroquine, azithromycin	Recovering by day 7 after the onset of weakness.
Scheidl et al. [47]	RT-PCR	No pathological findings	Negative *Campylobacter Jejuni* and Borrelia serology, negative ANA, anti-DNA, c-ANCA,p-ANCA	Increased total protein (140 g/L), albuminocytological dissociation	Demyelinating AIDP	Brain: NA Cervical spine: no pathological findings	IVIG 400 mg/kg (5 days)	None	Complete recovery
Sedaghat et al. [48]	RT-PCR + chest CT	Increased WBC 14.6 × 10^3^ (neutrophils 82.7%, lymphocytes 10.4%) and CRP	NA	NA	Motor sensory Axonal AMSAN	Brain: no pathological findings Spinal: two cervical intervertebral disc herniations	IVIG 400 mg/kg (5 days)	Hydroxychloroquine, lopinavir/ritonavir, azithromycin	Not reported
Sidig et al. [49]	RT-PCR + chest CT	NA	NA	None	Demyelinating AIDP	Brain: no pathological findings	NA	NA	Death after 7 days; because of progressive respiratory failure
Su et al. [50]	RT-PCR + chest X-ray	WBC 12,000 cells/µl	Negative anti- ganglioside GM1, GD1b and GQ1b antibodies, acetylcholine receptor binding, voltage-gated calcium channel, antinuclear and ANCA	Increased total protein (313 mg/dL), WBC: 1 cell	Demyelinating AIDP	NA	IVIG 2gm/kg (for 4 days)	None	On day 28 persistence of severe weakness
Tiet et al. [51]	RT-PCR	Elevated lactate on venous blood gas (3.3 mmo/L), mildly elevated CRP (20 mg/L). Normal WBC, sodium, potassium and renal function.	NA	Increased total protein (> 1.25 g/L), cell count 1x10^6^/L	Demyelinating AIDP	NA	IVIG 400 mg/kg/day (5 days)	None	Resolution of facial diplegia, improved upper and lower limbs weakness; able to mobilize unassisted 11 weaks after neurorehabilitation
Toscano et al. [52]	RT-PCR + Chest CT + serology	Lymphocytopenia, increased CRP, LDH, ketonuria	Negative anti-ganglioside antibodies	Day 2: normal total protein, no cells, negative SARS-CoV-2 PCR Day 10: increased total protein (101) mg/dl, cell count: 4/mm^3^, negative SARS-CoV-2 PCR	Axonal with sural sparing AMSAN	Brain: no pathological findings Spinal: Enhancement of caudal nerve roots	IVIG 400 mg/kg (2 cycles) + temporary mechanical non-invasive ventilation	Paracetamol	At week 4 persistence of severe UL weakness, dysphagia, and LL paraplegia
Toscano et al. [52]	RT-PCR (negative chest CT)	Lymphocytopenia; increased ferritin, CRP, LDH	NA	Increased total protein (123 mg/dl), no cells, negative SARS-CoV-2 PCR	Motor sensory axonal with sural sparing AMSAN	Brain: enhancement of facial nerve bilaterally Spinal: no pathological findings	IVIG 400 mg/kg	Amoxycillin	At week 4 improvement of ataxia and mild improvement of facial weakness
Toscano et al. [52]	RT-PCR + chest CT	Lymphocytopenia; increased CRP, LDH, ketonuria	Negative anti-ganglioside antibodies	Increased total protein (193 mg/dl), no cells, negative SARS-CoV-2 PCR	Motor axonal AMAN	Brain: no pathological findings Spinal: enhancement of caudal nerve roots	IVIG 400 mg/kg (2 cycles) + mechanical invasive ventilation	Azythromicin	ICU admission due to respiratory failure and tetraplegia. At week 4 still critical
Toscano et al. [52]	RT-PCR + serology (negative chest CT)	Lymphocytopenia; increased CRP, ketonuria	NA	Normal protein, no cells, negative SARS-CoV-2 PCR	Demyelinating AIDP	Brain: no pathological findings Spinal: no pathological findings	IVIG 400 mg/kg	None	At week 4 mild improvement in UL but unable to stand
Toscano et al. [52]	Chest CT + serology (negative RT-PCR in nasopharyngeal swab and BAL)	Lymphocytopenia; increased CRP, LDH	Negative anti-ganglioside antibodies; negative screening for *Campylobacter jejuni*, EBV, CMV, HSV, VZV, influenza, HIV	Normal total protein (40 mg/dL), white cell count 3/mm^3^; negative SARS-CoV-2 PCR	Demyelinating AIDP	Brain: NA Spinal: no pathological findings	IVIG 400 mg/kg + plasma exchange + mechanical invasive ventilation + enteral nutrition	None	At week 4 flaccid tetraplegia, dysphagia, ventilation dependent
Velayos Galán et al. [53]	RT-PCR + chest X-ray	NA	NA	NA	Demyelinating AIDP	NA	IVIG 400 mg/kg (5 days)	Hydroxychloroquine, lopinavir/ritonavir, amoxicillin, corticosteroids + low-flow oxygen therapy	NA
Virani et al. [54]	rt-pcr + chest mrt	WBC 8.6 × 10^3^; Hb 15.4 g/dl; PC 211 × 10^3^; procalcitonin: 0.15 ng/ml	NA	NA	NA	Brain: NA Spinal: no pathological findings	IVIG 400 mg/kg (5 days) + mechanical invasive ventilation (4 days)	Hydroxychloroquine 400 mg bid for first 2 doses, then 200 mg bid for 8 doses	At day 4 of IVIG: liberation from mechanical ventilation, resolution of UL symptoms, persistence of LL weakness. Sent to a rehabilitation facility
Webb et al. [55]	RT-PCR + chest X-ray + chest CT	Lymphopenia (0.9 × 10^9^/L), thrombocytosis (490 × 10^9^/L) raised CRP (25 mg/L)	Negative ANA, ANCA, anti-ganglioside antibodies, syphilis serology HIV, hepatitis B and hepatitis C	Increased total protein (0.51 g/L), normal glucose and cell count, negative SARS-CoV-2 PCR, negative viral PCR	Demyelinating AIDP	NA	IVIG 400 mg/kg/day (5 days) + Mechanical invasive ventilation	Co-amoxiclav	After 1 week in ICU: no oxygen requirement and ventilation
Zhao et al. [56]	RT-PCR + chest CT	WBC 0.52 × 10^9^; PC 113 × 10^9^/L	NA	Increased total protein (124 mg/dL), cell count 5 × 10^6^/L	Demyelinating AIDP	NA	IVIG (dosing not reported)	Arbidol, lopinavir/ ritonavir	At day 30 resolution of neurological and respiratory symptoms

AIDP, acute inflammatory demyelinating polyneuropathy; AMAN, acute motor axonal neuropathy; AMSAN, acute motor sensory axonal neuropathy; ANA, antinuclear antibodies; ANCA, anti-neutrophil cytoplasmic antibodies; BAL, bronchoalveolar lavage; CK, creatine kinase; CMV, cytomegalovirus; COPD, chronic obstructive pulmonary disease, COVID-19, coronavirus disease 2019; CRP, C-reactive protein; CSF, cerebrospinal fluid; CT, computed tomography; DM, diabetes mellitus; EBV, Epstein–Barr virus; ESR, erythrocyte sedimentation rate; F, female; GBS, Guillain–Barré syndrome; GGT, gamma-glutamyl transferase; GOT, glutamic oxaloacetic transaminase; GPT, glutamate pyruvate transaminase; Hb, haemoglobin; HIV, human immunodeficiency virus; HSV, herpex simplex virus; ICU, intensive-care unit; IL, interleukin; IVIG, intravenous immunoglobulins; IL, interleukin; LDH, lactate dehydrogenase; LL, lower limbs; M, male; MRI, magnetic resonance imaging; NA, not available; PC, platelet count; PCR, Polymerase Chain Reaction; SARS-CoV-2, severe acute respiratory syndrome coronavirus-2; TNF, tumor necrosis factor; UL, upper limbs; VDRL, Veneral Disease Research Laboratory; VZV, varicella-zoster virus; WBC, white blood cells; X-ray: radiography

^a^Time to Nadir refers to days elapsed between the onset of neurological symptoms and the development of the worst clinical picture when no progression was reported nadir was considered concomitant with GBS symptoms onset

^b^According to Brighton diagnostic criteria [66]

Interestingly, patients with no improvement or poor outcome (*n* = 19) showed a slightly higher (but not significant) frequency of clinical history and/or a radiological picture of COVID-19 pneumonia (14/19, 73.7%) compared to those with a favorable prognosis (29/48, 60.4%, *p* = 0.541). Moreover, the former group of patients was significantly older (mean 62.7 ± 17.8 years, *p* = 0.011), but with comparable distribution of sex (*p* = 0.622) and electrophysiological subtypes (*p* = 0.144) and similar latency between COVID-19 and GBS (*p* = 0.588) and nadir (*p* = 0.825), compared to the latter (mean age 51.8 ± 16.6 years). The same findings were confirmed even after excluding cases with no improvement from the analysis (to prevent a possible bias related to the short follow-up time).

## Discussion

COVID-19 pandemic prompts all efforts for the early recognition and treatment of its manifestations. In analogy to other viruses, belonging or not to the coronavirus family [63, 67], neurologic complications in COVID-19 are emerging as one of the most significant clinical chapters of this pandemic. In this regard, peripheral and central nervous system damage in COVID-19 has been postulated to be the consequence of two different mechanisms: 1) hematogenous (infection of endothelial cells or leucocytes) or trans-neuronal (via olfactory tract or other cranial nerves) dissemination to central nervous system in relation with viral neurotropism, and 2) abnormal immune-mediated response causing secondary neurological involvement [62, 68, 69]. The first mechanism is supposed to be responsible for the most common neurological symptoms developed by patients with COVID-19 (e.g., hypogeusia, hyposmia, headache, vertigo, and dizziness). In contrast, the second can lead to severe complications during or after the course of the illness, either dysimmune (e.g., myelitis, encephalitis, GBS) or induced by cytokine overproduction (hypercoagulable state and cerebrovascular events) [68, 69].

In the present systematic review, we reviewed clinical features, results of diagnostic investigations, and outcome in 73 cases of COVID-19-associated GBS spectrum [5–56].

In the present study, mean age at onset in patients with GBS largely overlapped that of classic COVID-19 subjects [70, 71]. However, pediatric cases with GBS have been increasingly reported in the literature [21, 27, 35, 41], suggesting that, with the spreading of the pandemic, a broader age range might be affected. Moreover, we found a higher prevalence of GBS in males compared to females, as previously reported for Zika virus–GBS [72]. This finding may also reflect the gender epidemiology of SARS-CoV-2. In this regard, males typically show a worse COVID-19 outcome compared to the females [70, 71], possibly due to a generally shorter life expectancy or to higher circulating Angiotensin-Converting-Enzyme 2 (ACE2) levels, the cellular receptor for SARS-CoV-2, in the former compared to the latter [71]. Moreover, given that GBS is a rare disease [57] the epidemiological distribution of the reported cases seems to reflect current worldwide outbreaks, with Europe being the “hottest” spot in March–May 2020 and USA together with Asia in the following period [73, 74]. On another issue, despite a few GBS cases seemed to have a para-infectious profile [10, 37, 38, 40, 55, 56] as described for Zika virus [75], all other reported patients developed neurological symptoms with a typical latency after COVID-19 (median time 14 days). This feature, together with the frequently reported negative nasopharyngeal swab at GBS onset [22, 24, 36, 44, 45, 52] and clinical improvement after IVIG therapy, seems to support the notion of a prominent post-infectious immune-mediated mechanism. However, in this context, the massive release of cytokines in COVID-19 may also contribute to the amplification of the dysimmune process underlying GBS [76, 77]. In this regard, the increase of blood inflammatory markers (e.g., CRP, IL-6, TNF-α, IL-1, etc.) in GBS tested cases may reinforce the hypothesis of a systemic inflammatory storm in COVID-19 [76, 77]. However, given the limited data, we could not perform an accurate analysis of the distribution and, eventually, prognostic value of inflammatory markers in COVID-19-associated GBS. Moreover, we cannot exclude that in cases with GBS developing before or together with COVID-19 symptoms, the disease might have progressed sub-clinically in the early phase to manifest afterwards with its typical systemic clinical picture. Indeed, two cases [10, 12**]**, who tested positive for SARS-CoV-2, never developed COVID-19 respiratory or systemic symptoms and one of them showed an asymptomatic pneumonia at chest-CT [12]. However, only more extensive epidemiological and translational studies, with the aim to compare the characteristics of GBS associated or not with COVID-19, could clarify these issues.

In our population, most common clinical manifestations and distribution of clinical variants resemble those of classic GBS confirming the predominance of the sensorimotor syndrome compared to MFS and other rare variants [57–59, 66]. Similarly, the results of CSF analysis reflected typical neurochemical findings in non-COVID-19 GBS. In the latter, elevated CSF proteins and pleocytosis were described in about 50–80% [57, 78] and 11–15% cases, respectively [58, 79, 80], largely overlapping with the percentages in our cohort. In this regard, the mostly normal cell count, together with the absence of SARS-CoV-2 RNA in all tested CSF samples [6–9, 12–14, 16, 21–24, 31, 33, 36, 42, 44, 52, 55], makes the possibility of a direct invasion from SARS-CoV-2 into the nerve roots with intrathecal viral replication less probable. However, a possible bias might rely on the lack of systematic data concerning the latency between symptom onset and CSF sampling in COVID-19 GBS cases. On another issue, in a further case of MFS associated withCOVID-19, who came to our attention, we observed the absence of intrathecal synthesis of SARS-CoV-2 antibodies together with a massive increase of CSF phosphorylated neurofilament heavy chain (pNfH) and serum neurofilament light chain (NfL) proteins, supporting the role of neurochemical markers as easily implementable tools for the detection of nervous system affection in COVID-19-related diseases [81, 82].

At variance with CSF findings, we found a discrepancy concerning MRI findings between classic GBS and COVID-19-related GBS. Specifically, while most cases of the former group showed typically spinal root enhancement at MRI [83], in the latter group, in analogy with Zika-associated GBS, the same finding was less frequently reported [84]. However, caution should be warranted in the interpretation of these results, given that MRI findings might have been underestimated, due to lack of a sufficient number of exams in the context of pandemic-imposed restrictions in the routine clinical setting.

Regarding the distribution of GBS electrophysiological variants, our analysis showed that COVID-19-associated GBS manifests prevalently with AIDP and, to a lesser extent, with AMSAN and AMAN, in line with classic GBS in Western countries [66, 85]. Conversely, the observation of positive anti-GD1b antibodies  in one COVID-19-related MFS patient and negative anti-ganglioside antibodies in other five cases appear in discordance with the high prevalence (≈ 90%) of anti-GQ1b antibodies among non-COVID-19 MFS cases [86], and may suggest different immune-mediated mechanisms. However, these results could not be generalized until a wider population would be tested.

In analogy to classic GBS, approximately one-fifth of COVID-19-associated GBS subjects required mechanical ventilation during hospitalisation [87]. In this regard, cases with no improvement or unfavorable outcome showed, in comparison to those with a good prognosis, an older age, confirming similar findings both in classic GBS [58, 88] and in COVID-19 [89], and a slightly higher frequency (without reaching a statistical significance) of past or concurrent COVID-19 pneumonia. However, given the short follow-up time in most cases, we could not reach a definite conclusion on the impact of past or concurrent COVID-19 restrictive syndrome due to pneumonia on the prognosis of GBS patients. Future prospective studies are needed to clarify this issue. Moreover, given that also preceding diarrhea (mostly caused by *Campylobacter Jejuni* infection) is a strong negative prognostic factor in classic GBS [57, 88], further prospective studies are needed to compare the severity of GBS related to COVID-19 to that associated with *C. jejuni*. Finally, in the context of respiratory failure and ventilation associated with COVID-19, the differential diagnosis should always take into consideration critical illness neuropathy and myopathy, which tend to develop later during the critical course [90]. Despite these findings, approximately one-third of COVID-19-related GBS patients showed no clinical and/or radiological evidence of pneumonia, providing evidence that GBS may also develop in the context of a paucisymptomatic or even asymptomatic COVID-19. However, given that among the GBS population only two asymptomatic COVID-19 patients were reported to date, we may speculate that, in most cases, a certain degree of lung injury (even minimal) or at least hematic dissemination (e.g., fever underlying significant viral load) is necessary to trigger the immuno-mediated process through lymphocytic recognition of self-antigens or molecular mimicry.

Major strengths of our review are the inclusion of a high number of patients, together with an in-depth analysis of the clinical and diagnostic features of COVID-19-associated GBS. We are aware that selection bias might have occurred, given that most reported cases to date have been described mostly in Europe (47 out of 73) and during COVID-19 highest spreading. Therefore, future extensive epidemiological studies are necessary to ascertain the nature of the association between COVID-19 and GBS (causal or coincidental). Moreover, we cannot exclude the possibility that at least some of the cases represent instances of CIDP, given the frequent absence of a follow-up longer than 2 months. On another issue, the low but possible evidence of an epidemiological link between vaccines and GBS development [57, 58] should aware the clinicians of the possible occurrence of GBS after COVID-19 vaccination in the long-term future.

In conclusion, based on the systematic review of 73 cases, we showed that the clinical picture of COVID-19-associated GBS seems to resemble that of classic GBS or Zika-associated GBS. Moreover, the chronological evolution, the response to IVIG, and the absence of SARS-CoV-2 RNA in CSF may suggest a prominent post-infectious immune-mediated mechanism rather than a para-infectious one. Although most cases were symptomatic for COVID-19, the preliminary report of a few patients without respiratory or systemic symptoms raises a significant healthcare issue, namely the importance of SARS-CoV-2 testing in all patients with suspected GBS during the pandemic, with the aim to provide an eventual rapid case isolation. Nevertheless, only further analyses on more comprehensive cohorts could help in clarifying better all these issues.
